# Relationship between 25(OH)D_2_, 25(OH)D_3_ and core symptoms in autism spectrum disorder

**DOI:** 10.3389/fped.2025.1622439

**Published:** 2025-08-22

**Authors:** Liu Jia, Du Hongfei, Zhao Deyun

**Affiliations:** ^1^ Department of Pharmacy, Children’s Hospital of Hebei Province, Shijiazhuang, Hebei, China; ^2^ Clinical Laboratory, Children’s Hospital of Hebei Province, Shijiazhuang, Hebei, China

**Keywords:** 25(OH)D_2_, 25(OH)D_3_, ASD, autism, vitamin D

## Abstract

**Background:**

This study intended to explore the levels of 25(OH)D_2_ and 25(OH)D_3_ in children with autism spectrum disorder (ASD) and analyzed the correlation between 25(OH)D_2_, 25(OH)D_3_ levels and ASD core symptoms, children development.

**Methods:**

A total of 208 children with ASD who were diagnosed in hospital from January 2021 to December 2023 were selected as the ASD group. 208 children for routine physical examination were selected as the control group. The contents of 25(OH)D_2_ and 25(OH)D_3_ in serum were detected by high-performance liquid chromatography tandem mass spectrometry (HPLC-MS/MS) method. The ASD group were assessed by Autism Behavior Checklist (ABC), Childhood Autism Rating Scale (CARS) and Gesell Development Schedule (GDS). Meanwhile, the correlations between the levels of 25(OH)D_2_, 25(OH)D_3_ and ABC, CARS, GDS in ASD children were analyzed.

**Results:**

The serum levels of 25(OH)D_2_ and 25(OH)D_3_ in the blood of the children in the ASD group were significantly lower than those in the control group (*P* < 0.050). There was no significant correlation between serum 25(OH)D_2_ levels and ABC, CARS, in children with ASD (*P* *>* 0.050). However, there were lower 25(OH)D_3_ levels were associated with more severe ASD symptoms (*P* < 0.050). The higher level of 25(OH)D_2_ was significantly correlated with lower adaptive behavior and personal-social of GDS (*P* < 0.050). The higher level of 25(OH)D_3_ was significantly correlated with higher adaptive behavior, fine motor and personal-social of GDS (*P* < 0.050).

**Conclusions:**

The levels of 25(OH)D_2_ and 25(OH)D_3_ in ASD children were significantly lower than those in healthy children. Besides, the study identified distinct roles for vitamin D isoforms including 25(OH)D_2_ and 25(OH)D_3_ in ASD pathophysiology. 25(OH)D_2_ showed selective impairments in adaptive behavior and personal-social behavior. 25(OH)D_3_ exhibited strong inverse correlations with symptom severity and positive associations with adaptive/fine motor/personal-social.

## Introduction

Autism spectrum disorders (ASD) was pervasive developmental disorder characterized in social communication disorder, repetitive stereotyped behavior, narrow interests and sensory abnormalities, which can affect children's brain development, immune system, gastrointestinal function and other aspects (1). Autism spectrum disorder had become an important public concern worldwide. There had been a great emphasis on the importance of early autism spectrum disorder diagnosis in large cities in China (2, 3). Incidence rate of autism spectrum disorder in the Avalon Peninsula was 1 case of autism spectrum disorder per 46 children. The prevalence in this region was also high when compared with other global populations (4). Although the prevalence of ASD was high, the pathophysiology was still unknown and heterogeneous. Vitamin D was a general name for steroid-like substances, which was a fat soluble vitamin. The important members of the family were vitamin D_2_ and vitamin D_3_ (5). Vitamin D_2_ or D_3_ binded to Vitamin D binding protein (VDBP) and were carried to the liver, where it was hydroxylated by several enzymes that had a 25-hydrolaxalse activity to form 25-hydroxyvitamin D [25(OH)D]. Subsequently, 25(OH)D was hydroxylated to 1,25-dihadroxyvitamin D [1,25(OH)_2_D], which was the biologically active form (6). In 2008, Cannell (7) was the first to proposed that vitamin D deficiency may play an important environmental risk factor in the etiology of ASD. Research had shown that vitamin D deficiency in pregnancy and early childhood could lead to the occurrence of ASD (8, 9). The sources of vitamin D_2_ and D_3_ were different. Vitamin D_2_ was only available from exogenous sources. However, vitamin D_3_ may be endogenous or exogenous. Besides, Vitamin D_2_ and vitamin D_3_ were structurally distinct. Specifically, the side chain of vitamin D_2_ contained a double bond between carbons 22 and 23 and a methylgroup oncarbon 24, both of which were absent from the side chain of vitamin D_3_. The two forms also had differing pharmacokinetics (10). Therefore, it was necessary to study the differential roles of vitamin D_2_ and D_3_ isoforms in ASD severity and neurodevelopment. This study aimed to elucidate serum 25-hydroxyvitamin D_2_ [25(OH)D_2_] and 25-hydroxyvitamin D_3_ [25(OH)D_3_] concentrations in ASD children compared to healthy controls, correlations with ASD symptom severity and neurodevelopmental outcomes.

## Methods

### Subjects

This study was a single-center research in northern China, latitude ∼38 °N. A total of 208 children diagnosed with Autism Spectrum Disorder (ASD) were selected as the ASD group, all of whom received treatment in the Psychological Behavior Department of our hospital from January 2021 to December 2023. The study group included 208 children aged between 1 and 5 years, comprising 112 boys and 96 girls. Simultaneously, the control group was established consisting of another set of 208 children aged between 1 and 5 years from the Children's Health Care Department of our hospital. These control subjects were selected through routine physical examinations and also included an equal distribution of genders: 112 boys and 96 girls. Importantly, members of the control group exhibited no symptoms indicative of ASD following preliminary diagnosis and assessment by qualified medical professionals. This study received approval from the Ethics Management Committee overseeing medical clinical trials at our hospital (NO: 202136), with informed consent obtained from the guardians of all participants.

The diagnosis of ASD in participating children adhered to criteria outlined in the Diagnostic and Statistical Manual of Mental Disorders—Fifth Edition (DSM-5) (11) published by the American Psychiatric Association, as well as utilizing the revised Autism Diagnostic Observation Schedule (12).

### Criteria

Inclusion criteria: 1. compliance with established diagnostic criteria for ASD; 2. voluntary participation in this study along with cooperation in completing relevant diagnostic assessments and scale scoring; 3. children diagnosed as ASD for the first time; 4. no history of vitamin D supplementation within three months prior to enrollment.

Exclusion criteria: Participants exhibiting other conditions that may confound this study will be excluded, including: 1. children whose main diagnosis was not autism; 2. children without perfect systematic examination; 3. children who cannot cooperate with the study.

### Detection method of 25(OH)D

Laboratory tests for 25(OH)D were performed by personnel from the pharmacological laboratory of the Department of Pharmacy. Two ml venous blood was collected, centrifuged for 6 min to obtain serum, and stored frozen in a refrigerator at −20 °C for subsequent test. The stability test confirmed that the samples should be frozen (−20 °C)for no more than 1 month. The half-life of vitamin D was 24 h (13), while the half-life of 25(OH)D was 3 weeks, and the half-life of 1,25-(OH) 2-D was 4 h (14). Due to the long half-life of 25(OH)D, the serum concentrations of 25-hydroxyvitamin D_2_ [25(OH)D_2_] and 25-hydroxyvitamin D_3_ [25(OH)D_3_] were used to represent the serum concentrations of vitamin D_2_ and vitamin D_3_ in the actual detection process respectively. The contents of 25(OH)D_2_ and 25(OH)D_3_ in serum were detected by high-performance liquid chromatography tandem mass spectrometry (HPLC-MS/MS) method.

Samples were run on a Jasper™ HPLC system with a Triple Quad™ 4500MD mass spectrometer from AB SCIEX (Framingham, MA USA). Analyst 1.6.3 and MultiQuant™ MD 3.0.2 software were used for instrument control, data acquisition, and data processing. Chromatographic separation was performed on a Kinetex® EVO C18, 2.600 µm, 30 × 2.100 mm HPLC column (Phenomenex, Torrance, CA, USA) using a binary gradient elution 0–0.500 min, 80%B; 0.500–2.000 min 98%B; 2.000–5.000 min 98%B; 5.000–6.000 min 80%B; 6.000–7.000 min. The mobile phase (A) and (B) were from Shandong Yingsheng Biotechnology Co., Ltd and the flow rate was 0.550 ml/min. The injection volume was 5 µl. The column oven was kept at 45 °C. The autosampler temperature was maintained at 5 °C.

Mass spectrum acquisition was performed in positive electrospray ionization mode with multiple-reaction monitoring (MRM). Mass parameters were set: temperature, 600 °C; curtain gas, 35 psi; ion spray voltage, 5,500 V; ion source gas 1, 50 psi; and ion source gas 2, 55 psi. Dwell time was set at 30 ms for each compound. The declustering potential, entrance potential, and collision energy were optimized at 92 V, 10 V, and 28 eV, respectively. LC-MS/MS data were analyzed using Analyst 1.6.3 software.

### Observations

The autism behavior checklist (ABC) (15) contained 57 items, which encompassed five factors, such as sensory (S), relating (R), body and object use (B), language (L), and social and self-help skills (V) (16). In 1989, Professor Yang of Beijing Medical University introduced and revised the score, which were used to screen autistic children. Besides, Childhood Autism Rating Scale (CARS) listed 15 items, and each was graded from 1 to 4 (17). ASD severity was evaluated using ABC and CARS. Higher scores of ABC and CARS scores represented more serious autistic symptoms. Intellectual and behavioral development in ASD children was assessed via the revised Gesell Developmental Schedule (GDS). The evaluation results were presented in the form of a developmental quotient (DQ). GDS covered five sectors, including adaptive behavior, gross motor, fine motor, language, and personal-social behavior (18). The higher development quotient (DQ) scores, the closer the developmental level was to or exceeds that of normal children. During the assessment of ABC, CARS and GDS for children with autism, it was found that many children could not socialize normally, resulting in that many children did not complete all assessment of ABC, CARS and GDS. Many patients had selectively completed one, two or three assessments according to individual differences. In this study, the specific number of participants in each project were as follows: 74 cases were scored with the ABC; 74 cases were scored with the CARS; 60 cases were scored with the GDS. These cases were strictly screened according to the above criteria by two workers. The two doctors took up their posts after unified training. Besides, they were specially responsible for the evaluation of children and had rich experience.

### Data analysis

SPSS 21.0 software was used to analyze the data. Normally distributed data were expressed as mean ± standard deviation (SD). Numerical variables without normal distribution were shown as mean ± SD, as well as median values and interquartile range (IQR). The difference of measurement data was detected by *t* test. If the data were non-normal distribution, *Mann–Whitney U test* was used. *Pearson correlation test* was used to test the correlation of parameters. All analyses in this study were bilateral tests. Differences were deemed statistically significant when *p* < 0.050.

### Role of the funding source

This research project was derived from Medical Science Research Project of Hebei (NO: 20220772). The Health Commission of Hebei Province had approved this project. Guidance on policies and directional resources were provided. The corresponding authors were responsible for all aspects of the study. The final version was approved by all authors.

## Results

### Comparison of serum levels of 25(OH)D_2_ and 25(OH)D_3_ between ASD groups and control groups

**Figure 1:**
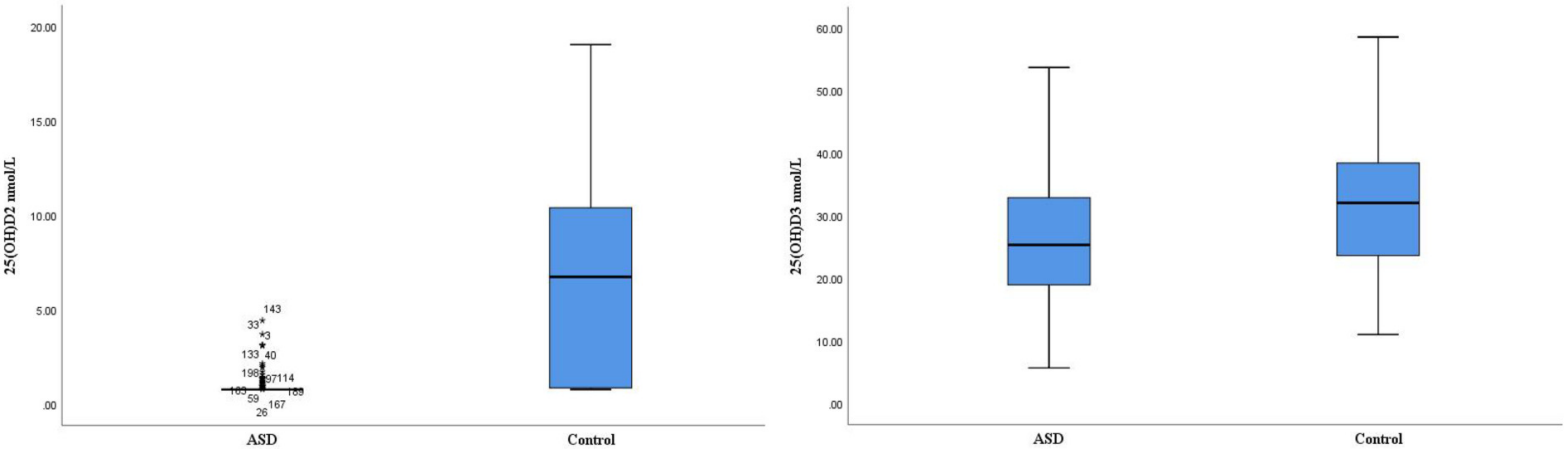
The levels of 25(OH)D_2_ and 25(OH)D_3_ between ASD groups and control groups.

The serum levels of 25(OH)D_2_ and 25(OH)D_3_ were assessed in children with ASD group compared to healthy controls. Notably, the level of 25(OH)D_2_ in the blood of children within the ASD group was significantly lower than that observed in the control group [(0.913 ± 0.419) vs. (6.841 ± 5.230) nmol/L, *P* = 0.001]. Similarly, the level of 25(OH)D_3_ in the ASD group was significantly reduced compared to controls [(26.862 ± 8.200) vs. (31.953 ± 9.400) nmol/L, *P* = 0.015] (Table 1, Figure 1).

**Table 1:** Distribution characteristics of research objects.

Item	ASD (*N* = 208)	Control (*N* = 208)	*P* value
Age (*x* ± *s*)	3.791 ± 0.230	3.210 ± 0.573	
Boy	112	112	
Girl	96	96	
ABC	74	0	
CARS	74	0	
GDS	60	0	
25(OH)D_2_ ng/ml			***0.001***
Mean ± SD	0.913 ± 0.419	6.841 ± 5.230	
Median	0.780	6.735	
IQR	0.000	9.610	
25(OH)D_3_ ng/ml			***0.015***
Mean ± SD	26.862 ± 8.200	31.953 ± 9.400	
Median	25.320	32.020	
IQR	14.000	14.860	

Meaning of the bold values were the *P* values.

### Correlation analysis of 25(OH)D2 and 25(OH)D3 levels with ABC scores in children with ASD

*Pearson linear correlation analysis* was conducted to examine the relationship between serum levels of 25(OH)D_2_, 25(OH)D_3_ and ABC scores among a subset of 74 ASD patients (34 boys and 40 girls). No statistically significant correlation was found between the level of 25(OH)D_2_ and ABC scores (*r* = −0.027, *P* = 0.872). In contrast, the lower 25(OH)D_3_ levels were associated with higher ABC scores. (*r* = −0.35, *P* = 0.034).

### Correlation analysis of 25(OH)D2 and 25(OH)D3 levels with CARS scores in children with ASD

*Pearson linear correlation analyses* were performed on serum levels of both 25(OH)D_2_, 25(OH)D_3_ concerning CARS scores among another set of ASD patients (44 boys and 30 girls). The results indicated no significant association between levels of serum 25(OH)D_2_ and CARS scores (*r* = 0.200, *P* = 0.236). However, a notable correlation was identified for the lower levels of serum 25(OH)D_3_ with higher CARS scores (*r* = −0.453, *P* = 0.048) demonstrating statistical significance.

### Correlation analysis of 25(OH)D2 and 25(OH)D3 levels with Gesell dimensions in children with ASD

Additionally, an analysis involving *pearson linear correlation* examined serum concentrations of both forms of 25(OH)D_2_, 25(OH)D_3_ alongside GDS assessments from a sample size comprising 60 patients with ASD (34 boys and 26 girls). As presented in Table 2, there existed a significant negative correlation that the higher level of 25(OH)D_2_ was significantly correlated with lower adaptive behavior and personal-social of GDS (*P* < 0.050). Such significance was not noted regarding gross motor, fine motor or language dimensions (*P* *>* 0.050). Conversely, 25(OH)D_3_ demonstrated positive correlations that the higher level of 25(OH)D_3_ was significantly correlated with higher adaptive behavior, fine motor and personal-social of GDS (*P* < 0.050). But it showed no associations with gross motor or language dimensions in Table 3 (*P* *>* 0.050).

**Table 2:** The relationship between 25(OH)D_2_ and GDS in ASD children.

Item	Adaptability	Gross motor	Fine motor	Language	Personal-social
*r*	−0.570	−0.326	−0.347	−0.386	−0.469
*P*	0.009	0.161	0.134	0.093	0.037

**Table 3:** The relationship between 25(OH)D_3_ and GDS in ASD children.

Item	Adaptability	Gross motor	Fine motor	Language	Personal-social
*r*	0.539	0.268	0.467	0.306	0.472
*P*	0.014	0.253	0.038	0.190	0.036

## Discussion

Vitamin D receptor (VDR) were found in many cells, not just those involved with calcium and phosphate homeostasis. Vitamin D exerted its effects on target cells through both genomic and non-genomic mechanisms. Some studies had confirmed that vitamin D deficiency was implicated in the etiology of AS (19). Deficiency during pregnancy could disrupt normal brain development process. Study indicated that blood-brain cross-talk was crucial for ASD pathophysiology. Besides, the brain may sense peripheral system changes through exosomes (20). Another study confirmed the novel role of exosomes as carriers of miRNAs with the ability to cross the blood-brain barrier and unique expression profiles, offering new possibilities for diagnostic and therapeutic interventions in ASD (21). Furthermore, vitamin D deficiency can lead to an imbalance in neurotransmitters, diminish the body's and brain's antioxidant capacity, and alter immunological responses (22, 23).

It has been established that the molecular structures of vitamin D_2_ and vitamin D_3_ differed significantly. It was the first study to investigate the relationship between vitamin D_2_, vitamin D_3_ in ASD. In this research, the levels of 25(OH)D_2_ and 25(OH)D_3_ were measured using HPLC-MS/MS, a method known for its high sensitivity and specificity. This approach offered certain methodological advantages over other techniques. The findings revealed that the levels of 25(OH)D_2_ and 25(OH)D_3_ were significantly lower in children with ASD compared to their healthy counterparts. Furthermore, this study analyzed the correlation between 25(OH)D_2_ and 25(OH)D_3_ levels and the severity of ASD symptoms. The results indicated that the higher level of 25(OH)D_2_ was significantly correlated with lower adaptive behavior and personal-social of GDS. Conversely, 25(OH)D_3_ demonstrated associations not only with intellectual and behavioral development related to adaptability but also with fine motor and personal-social abilities in these children; Additionally, it was linked to symptom severity. Studies had confirmed that two forms of vitamin D exhibited distinct abilities to elevate serum levels of 25-hydroxyvitamin D [25(OH)D]. Specifically, studies corroborated that increases in serum 25(OH)D levels attributable to administration of vitamin D_3_ were nearly equivalent to those achieved by administering double the amount of vitamin D_2_ (24). Besides, the concentration of the vitamin D_2_ metabolite bound to VDBP in plasma was lower than that associated with vitamin D_3_ (25). Administration of vitamin D_2_ could reduce 25-hydroxylation of vitamin D_3_ and 1-αhydroxylation of 25(OH)D_3_, while increase 24R-hydroxylation of 25(OH)D_3_ (10). Another research indicated that the efficacy of vitamin D_3_ surpassed that of vitamin D_2_ (26).

This research had certain limitations. As a single—center study, it was likely to potentially introducing regional bias. Furthermore, confounding factors, including dietary vitamin D intake and sunlight exposure, were not accounted for. In addition, not all of the sample of children with ASD completed all evaluations of the proposed questionnaires, which could lead to a small sample size for some evaluations, such as the sample size of the GDS was relatively small. Therefore, this discrepancy may have limited the results obtained to the correlation between vitamin D levels and severity of the pathology. Although this study had a small sample size, it provided novel insights into the role of vitamin D in relation to ASD.

## Data Availability

The raw data supporting the conclusions of this article will be made available by the authors, without undue reservation.
